# Supplementary material for: On the importance of assessing topological convergence in Bayesian phylogenetic inference
Source: ArXiv. 2024 Aug 19:arXiv:2402.11657v2. Preprint. [Version 2] (PMC11383445)
Supplement: Supplement 1 [file NIHPP2402.11657v2-supplement-1.pdf]

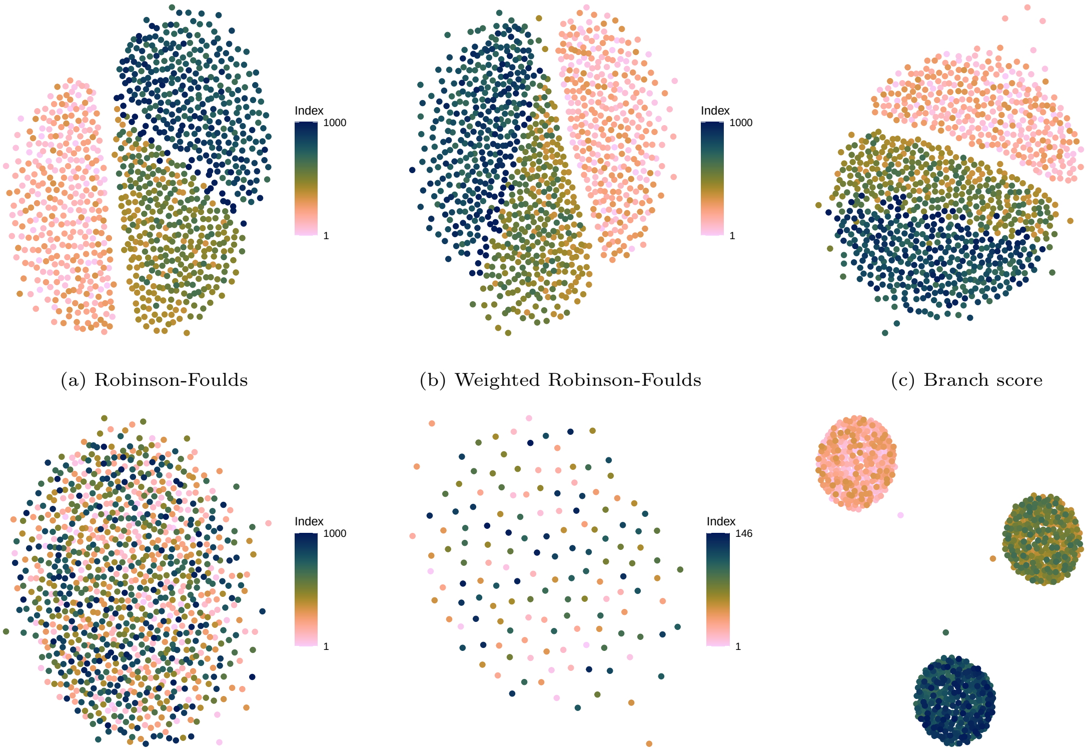

(a) Robinson-Foulds (b) Weighted Robinson-Foulds (c) Branch score

(d) Path difference (e) Kendall-Colijn ($\lambda = 0$) (f) aSPR

Fig. S1. EBOV networks for all phylogenetic distance metrics. For the Kendall-Colijn distance, the full sample was downsampled to 146 trees equally spaced between tree 1 and tree 1000, as computing pairwise distances for 1000 trees was not feasible.

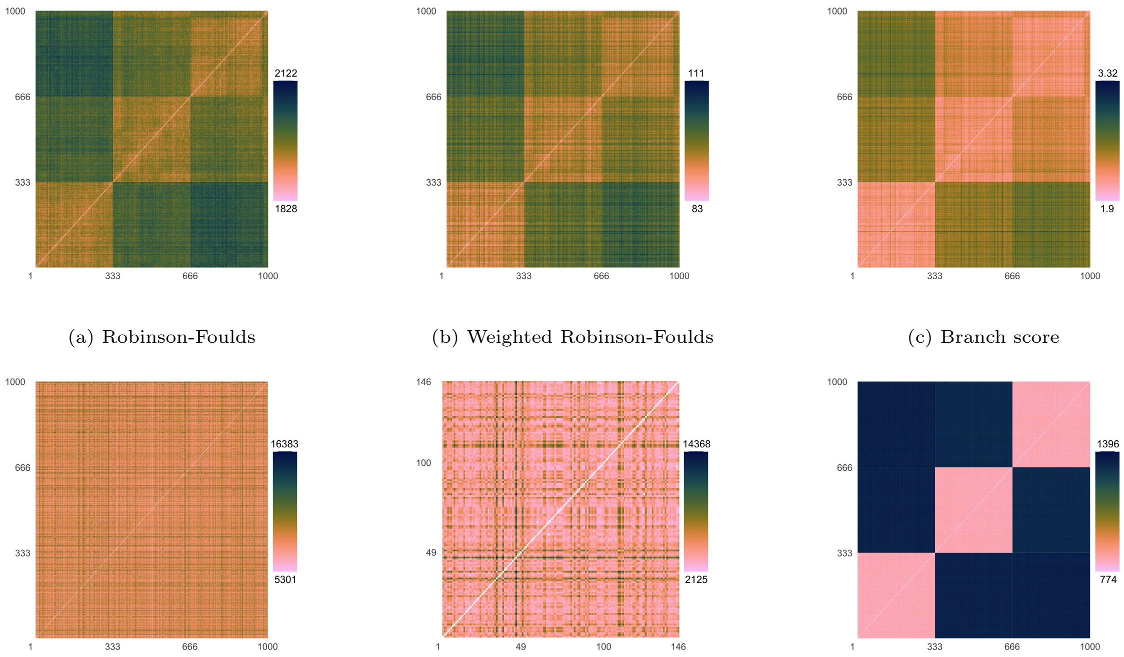

(a) Robinson-Foulds (b) Weighted Robinson-Foulds (c) Branch score

(d) Path difference (e) Kendall-Colijn ($\lambda = 0$) (f) aSPR

Fig. S2. EBOV heatmaps for all phylogenetic distance metrics. For the Kendall-Colijn distance, the full sample was downsampled to 146 trees equally spaced between tree 1 and tree 1000, as computing pairwise distances for 1000 trees was not feasible.

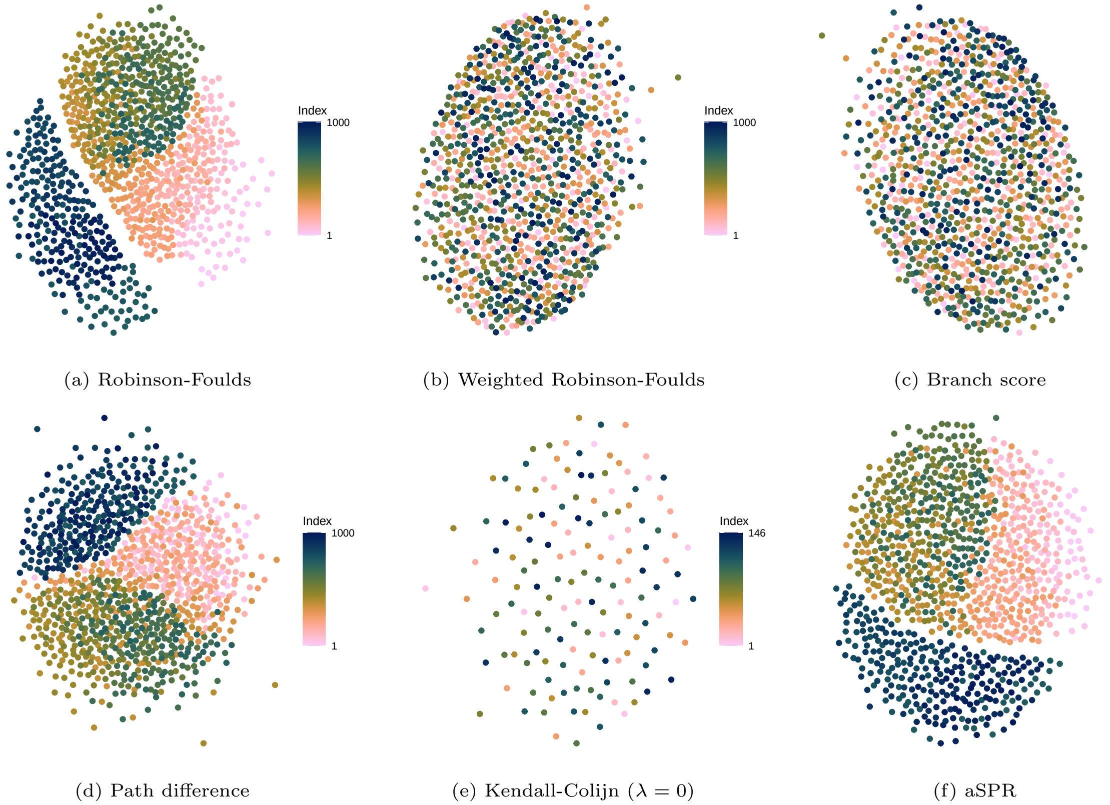

Fig. S3. HIV networks for all phylogenetic distance metrics. For the Kendall-Colijn distance, the full sample was downsampled to 146 trees equally spaced between tree 1 and tree 1000, as computing pairwise distances for 1000 trees was not feasible.

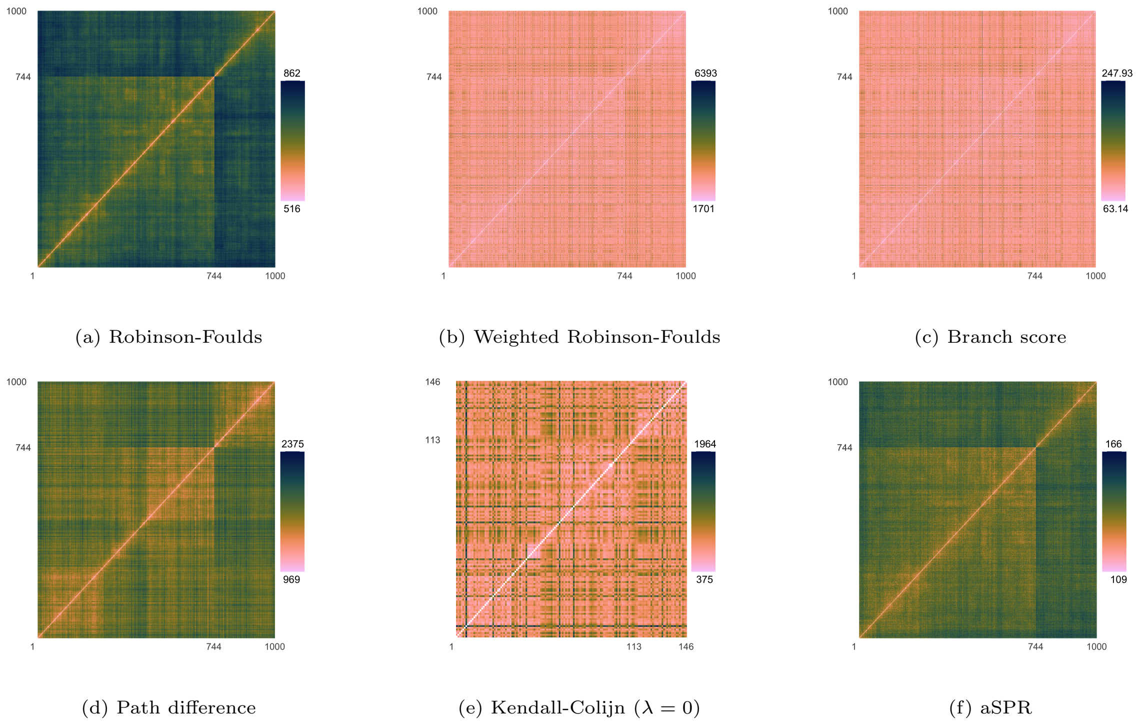

Fig. S4. HIV heatmaps for all phylogenetic distance metrics. For the Kendall-Colijn distance, the full sample was downsampled to 146 trees equally spaced between tree 1 and tree 1000, as computing pairwise distances for 1000 trees was not feasible.

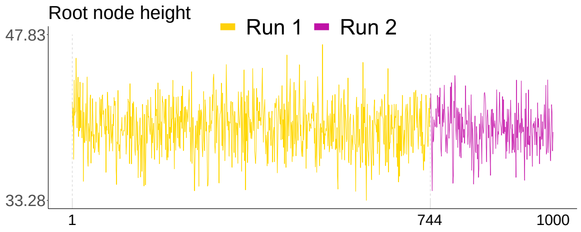

(a) Root node height (HIV trees) — $ESS = 840$

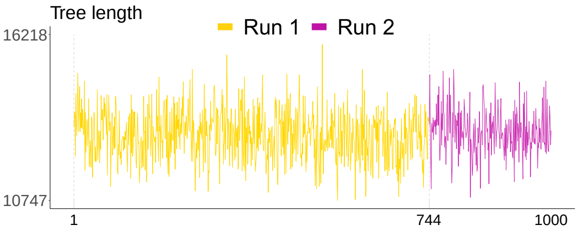

(b) Tree length (HIV trees) — $ESS = 687$

Fig. S5. Trace plots for the root node height and tree length for the HIV samples. Tree length refers to the sum of all tree branches, and is thus a statistic closely related to the topology of the tree. Neither of these statistics show a discrepancy between the two runs. Note that the shape of the traces are extremely similar to each other (although not identical), which could again be related to the star-like shape of HIV phylogenies.

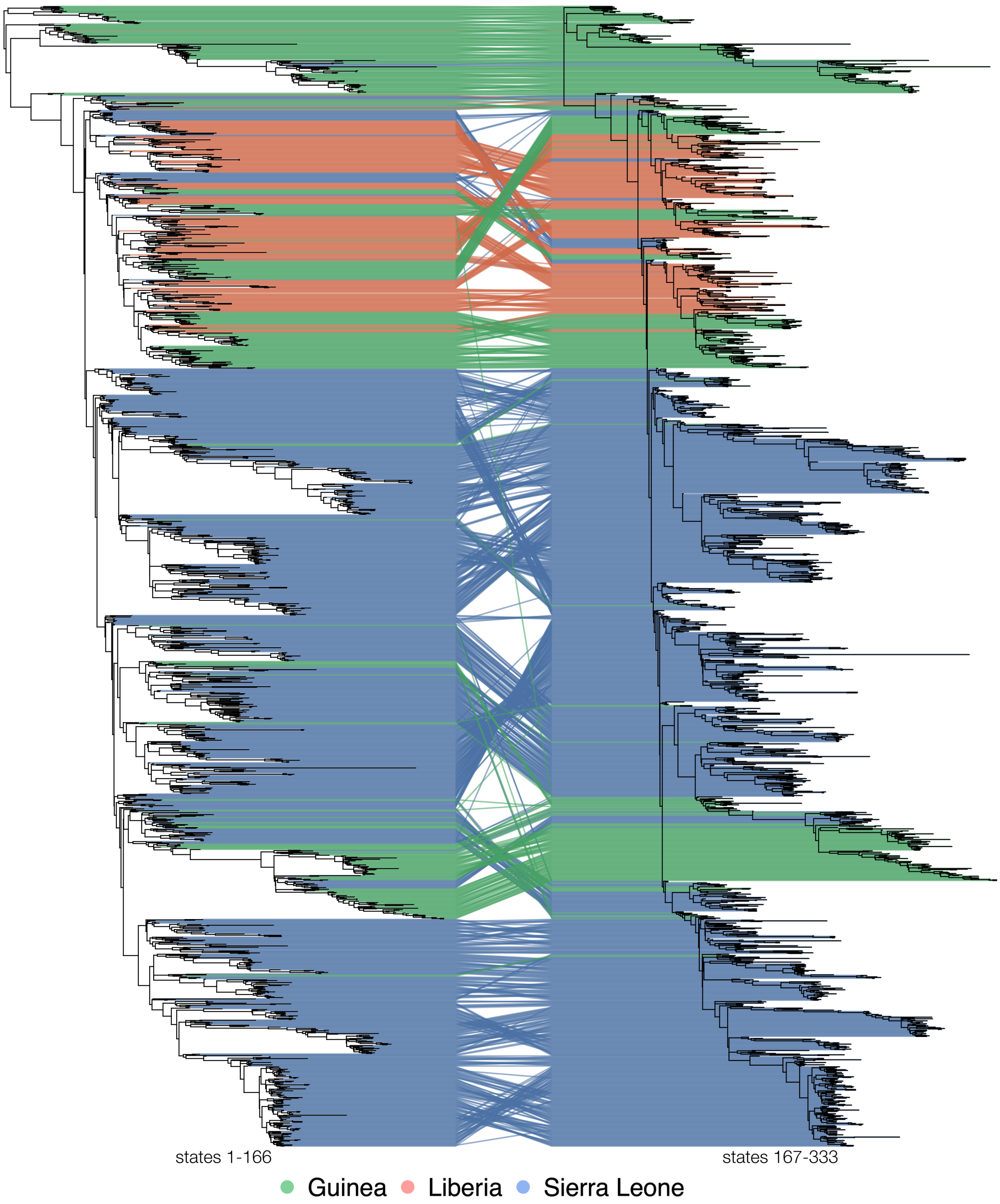

Fig. S6. Tanglegram of the MCC trees of the first and second half of the first EBOV run. The tips of the trees are connected to each other by lines, coloured by the country of origin. Disagreement between the subsamples regarding the location of several clades is apparent by the fact that the lines connecting the tips of these clades are not parallel.

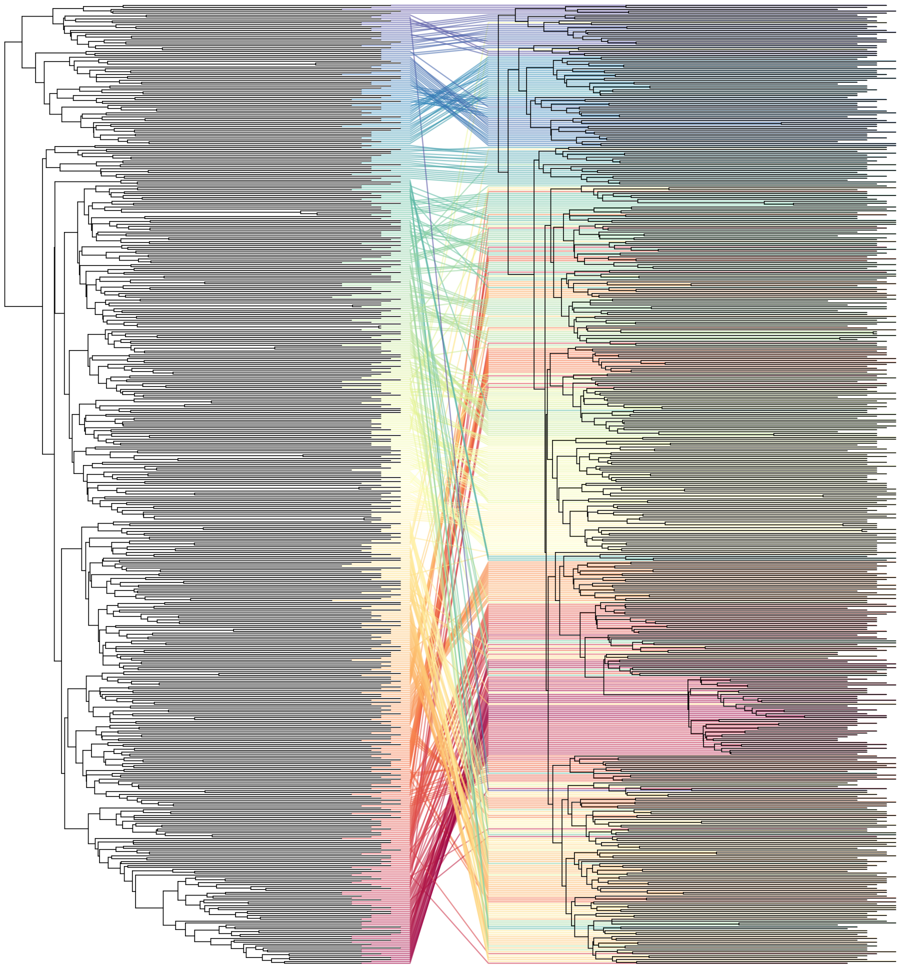

Fig. S7. Tanglegram of the MCC trees of the first and second half of the first HIV run. The tips of the trees are connected to each other by lines, coloured by position in the first MCC tree. Disagreement between the subsamples regarding the location of several clades is apparent by the fact that the lines connecting the tips of these clades are not parallel.

Table S1. Root height and tree length ESS for EBOV subsamples.

Subsample	Root Height	Tree Length
1-333	204	113
334-666	173	140
667-1000	309	143
1-1000	726	20

Table S2. Root height and tree length ESS for HIV subsamples.

Subsample	Root Height	Tree Length
1-744	609	366
745-1000	221	220
1-1000	840	687
